# Global Transcriptomic Analysis and Function Identification of Malolactic Enzyme Pathway of *Lactobacillus paracasei* L9 in Response to Bile Stress

**DOI:** 10.3389/fmicb.2018.01978

**Published:** 2018-08-23

**Authors:** Xiayin Ma, Guohong Wang, Zhengyuan Zhai, Pengyu Zhou, Yanling Hao

**Affiliations:** ^1^Beijing Advanced Innovation Center for Food Nutrition and Human Health, College of Food Science and Nutritional Engineering, China Agricultural University, Beijing, China; ^2^Key Laboratory of Functional Dairy, Co-constructed by the Ministry of Education and Beijing Municipality, China Agricultural University, Beijing, China

**Keywords:** *Lactobacillus paracasei* L9, bile stress, RNA-seq, malolactic enzyme pathway, alkalinization, membrane vesicles, membrane balance

## Abstract

Tolerance to bile stress is crucial for *Lactobacillus paracasei* to survive in the intestinal tract and exert beneficial actions. In this work, global transcriptomic analysis revealed that 104 genes were significantly changed (log_2_FoldChange > 1.5, *P* < 0.05) in detected transcripts of *L. paracasei* L9 when exposed to 0.13% Ox-bile. The different expressed genes involved in various biological processes, including carbon source utilization, amino acids and peptide metabolism processes, transmembrane transport, transcription factors, and membrane proteins. It is noteworthy that gene *mle*S encoding malolactic enzyme (MLE) was 2.60-fold up-regulated. Meanwhile, L-malic acid was proved to enhance bile tolerance, which could be attributed to the intracellular alkalinization caused by MLE pathway. In addition, membrane vesicles were observed under bile stress, suggesting a disturbance in membrane charge without L-malic acid. Then, genetic and physiological experiments revealed that MLE pathway enhanced the bile tolerance by maintaining a membrane balance in *L. paracasei* L9, which will provide new insight into the molecular basis of MLE pathway involved in bile stress response in Lactic acid bacteria.

## Introduction

*Lactobacillus paracasei* is a common constituent of inhabitants in the human and animal gut, and is extensively used in the food industry as a starter culture for dairy fermentation (Sisto and Lavermicocca, 2012; Di Renzo et al., 2018; Stefanovic et al., 2018). Furthermore, some strains of *L. paracasei* have attracted additional interest as health-promotion probiotics (De Vrese and Schrezenmeir, 2008). To exert their beneficial effects, high survival rate and stable colonization in the human gastrointestinal tract (GIT) are crucial for *L. paracasei* strains. However, during digestion, *L. paracasei* suffers from various challenges in the GIT, such as acidic pH in the stomach and bile salts in the intestine (Corcoran et al., 2008; Bove et al., 2013). Bile salts are known as detergent-like biological compounds with antimicrobial activity, which are capable of disrupting the lipid bilayer structure of cellular membranes. Damage due to bile salts includes protein misfolding and denaturation, DNA or RNA damage, and intracellular acidification (Begley et al., 2005). Therefore, tolerance to bile stress is essential for *L. paracasei* to survive and colonize in the GIT.

A number of studies have explored the mechanism of bile resistance in members of genus *Lactobacillus*, such as *Lactobacillus plantarum* (Hamon et al., 2011b), *Lactobacillus rhamnosus* (Koskenniemi et al., 2011), and *Lactobacillus casei* (Hamon et al., 2011a; Alcantara and Zuniga, 2012). Generally, bile salts hydrolases (BSHs) contribute to bile tolerance by hydrolyzing the protonated conjugated bile salts into unconjugated counterparts (Smet et al., 1995). On the other hand, efflux pumps are a common mechanism in lactobacilli, and this could extrude the bile salts from the cell, such as the MDR transporters in *L. acidophilus* (Pfeiler and Klaenhammer, 2009). In addition, changes in fatty acid metabolism have been described under bile exposure, which could alter the lipid composition of bacterial membranes to reduce bile diffusion (Taranto et al., 2003). Probiotic bacterial species differ from each other in their resistances to bile salts, and even within one species, the strain-specific variation in bile salt tolerance is remarkable. For example, the gene encoding glucosamine-6-phosphate deaminase (NagB) involved in *N*-acetylneuraminate degradation was 7.54-fold up-regulated in detected transcripts in *L. casei* BL23. However, this gene was down-regulated in other *L. casei* strains under bile stress (Hamon et al., 2011a; Alcantara and Zuniga, 2012).

*Lactobacillus paracasei* L9 (CGMCC No. 9800) originated from healthy human intestine (Yang et al., 2015). Previous research has revealed the health-promoting effects of this strain, such as regulating host immunity (Yang et al., 2015) and preventing intestinal damage (Pan et al., 2014). In this study, RNA-Seq was performed to analyze the global bile stress response and resistance mechanisms in *L. paracasei* L9. Transcriptomic data revealed that differentially expressed genes (DEGs) participate in various biological processes, such as carbohydrate and amino acid metabolism, transcriptional regulation, fatty acid biosynthesis, and general stress response. It is worth noting that there was an increase in detected transcripts for malolactic enzyme (MLE) pathway genes. The MLE pathway is a secondary fermentation during wine making, in which the dicarboxylic acid L-malate is converted into L-lactate and carbon dioxide by the MLE (Caspritz and Radler, 1983). The MLE pathway enhanced bacterial viability under environmental stress conditions in *Streptococcus mutans*, such as low pH (Lemme et al., 2010), oxidative stress and starvation (Sheng et al., 2010). In the present work, physiological and genetic experiments were performed to demonstrate that MLE enhanced the bile tolerance by maintaining a membrane balance in *L. paracasei* L9.

## Materials and Methods

### Bacterial Strains, Plasmids, and Growth Conditions

Bacterial strains and plasmids used in this study are listed in the **Supplementary Table S1**. *L. paracasei* L9 was cultured in de Man-Rogosa-Sharpe (MRS) medium anaerobically at 37°C. Growth assays were carried out in chemically defined medium (CDM; Zhou, 2010). CDMM was CDM supplemented with 2.5 mg/mL L-malic acid. *Escherichia coli* was grown aerobically at 37°C in Luria-Bertani (LB) medium with shaking at 200 rpm. When required, media were supplemented with antibiotics at the following concentrations: 10 μg/mL erythromycin for *L. paracasei* L9, 100 μg/mL ampicillin for *E. coli* DH5α. Ox-bile (Sigma, St. Louis, MO, United States) was added at different concentration as needed. To investigate the influence of L-malic acid on the growth of cells under bile stress, overnight cultures of *L. paracasei* strains were 1% inoculated in CDM and CDMM containing 0.2% Ox-bile. And the OD_600_ was determined at 2 h intervals. All results were obtained from at least three independent experiments.

### Transcriptomics

Overnight cultures of L9 were inoculated at 1% in 50 mL MRS with or without 0.13% Ox-bile, and then incubated anaerobically at 37°C. Cells were harvested when cultures reached mid-exponential phase (OD_600_ of 0.6, 3–4 × 10^7^ cfu/mL). Three independent biological replicates were performed in this study. Total RNA isolation was performed with TRIzol reagent (Invitrogen, Carlsbad, CA, United States) according to the manufacturer’s instructions. For RNA sample preparation, 3 μg RNA per sample was used as input material. Sequencing libraries were generated using NEBNext^®^ Ultra™ Directional RNA Library Prep Kit for Illumina^®^ (NEB, United States) following the manufacturer’s recommendations and index codes were added to attribute sequences. Library fragments were then purified using AMPure XP system (Beckman Coulter, Beverly, MA, United States), in order to preferentially select cDNA fragments of 150–200 bp in length. Size-selected and adaptor-ligated cDNA was then treated with 3 μl USER Enzyme (NEB, United States) at 37°C for 15 min followed by 5 min at 95°C. PCR was then performed using Phusion High-Fidelity DNA polymerase with Universal PCR primers and Index (X) Primer. Finally, products were purified (AMPure XP system) and library quality was assessed on the Agilent Bioanalyzer 2100 system. The clustering of the index-coded samples was performed on a cBot Cluster Generation System using TruSeq PE Cluster Kit v3-cBot-HS (Illumia) according to the manufacturer’s instructions. After cluster generation, the library preparations were sequenced on an Illumina Hiseq platform and paired-end reads were generated.

Raw data (raw reads) of fastq format were firstly processed through in-house perl scripts. All sequenced reads were aligned to the genome of *L. paracasei* L9 with GenBank Accession No. NZ_CP012148.1. HTSeq v0.6.1 was used to count the reads numbers mapped to each gene. Differential expression analysis of two groups was performed using the DESeq R package (1.18.0). The resulting *P*-values were adjusted using Benjamini and Hochberg’s approach for controlling the false discovery rate. Genes with an adjusted *P*-value < 0.05 found by DESeq were assigned as differentially expressed.

### DNA Manipulation Techniques

Chromosomal DNA from *L. paracasei* was extracted using TIANamp Bacteria DNA Kit according to the manufacturer’s instructions (Tiangen, Beijing, China). Lysis of *L. paracasei* was performed by adding 30 mg/mL lysozyme dissolved in TES buffer (50 mM Tris-HCl, 1 mM EDTA, 25% sucrose; pH 8.0), and incubating the suspension at 37°C for 1 h. Miniprep plasmid isolation from *E. coli* was performed using the Plasmid Mini Kit I (OMEGA Bio-tek, Inc., Doraville, GA, United States). PCR was carried out using Q5^®^High-Fidelity DNA Polymerase (NEB, United States). Primers used in PCR reactions are listed in **Supplementary Table S2**. Restriction endonuclease digestions were conducted according to the supplier’s instructions (Takara, Dalian, China). DNA ligation was performed using the T4 DNA Ligation Kit (Thermo Fisher Scientific, Beijing, China). Plasmids were introduced into *E. coli* DH5α using standard heat shock transformation (Tiangen, Beijing, China). Plasmids were introduced into *L. paracasei* L9 by electroporation as described previously (Aukrust et al., 1995). DNA sequencing was performed with the BigDye Terminator cycle sequencing kit (Sangon, Beijing, China) and the results were further analyzed with the DNAMAN software package (Version 6.0.3, Lynnon Biosoft, Canada).

### Insertional Inactivation of the Gene *mle*S

In order to study the role of MLE pathway in bile stress, a *mle*S mutant of *L. paracasei* L9 was obtained by a single crossover homologous recombination as shown in **Supplementary Figure S3**. A 650-bp internal region of the *mle*S gene (300–950 bp) was chosen as a homologous sequence and amplified using the primers pair LPL9_M0797F and LPL9_M0797R with flanking *Xba*I and *Eco*RI sites, respectively. The resulting PCR product was restriction enzyme digested and ligated into the corresponding restriction sites of the suicide plasmid pUC19EM (Yang et al., 2017). The recombinant plasmid, designated pUCmleS, was then introduced into *L. paracasei* L9 by electroporation, and the recombinant strain were cultivated on MRS solid medium containing erythromycin. As the pUCmleS could not replicate in *L. paracasei* L9, the erythromycin selection pressure resulted the integration of the plasmid into the *mle*S gene region of the *L. paracasei* L9 genome. The resulting mutant was designated L9mleS^-^. To confirm the integration of pUCmleS into the correct genome locus, PCR was performed with forward primer EM-F and reverse primer 0797D-R, which were designed according to the DNA sequence of the erythromycin resistance gene (GenBank Accession No. KM017875.1) and the downstream sequence of LPL9_0797, respectively.

### Field-Emission Scanning Electron Microscopy

Cells from *L. paracasei* L9 in CDM with or without 0.2% Ox-bile and cells in CDMM with 0.2% Ox-bile were collected at 12 h and fixed with 2.5% glutaraldehyde in phosphate buffer solution (PBS) at 4°C. Samples were dehydrated in a graded ethanol series, and then transferred into the chamber of the critical point dryer (CPD 030 critical point dryer; Bal-Tec) to replace the ethanol with liquid CO_2_. Glass samples were sputter-coated with platinum layer to be imaged by a field-emission scanning electron microscopy (FESEM, Hitachi SU8010, Japan) with an acceleration voltage of 5 kV.

### Hydrophobicity Assays

The microbial adhesion to solvents (MATS) method was employed for the evaluation of the hydrophobic/hydrophilic character of the cell surface of strains of *L. paracasei* (Bellon-Fontaine et al., 1996). Cells from *L. paracasei* L9 and L9mleS^-^ in CDMM were harvested at 12 h by centrifugation (7000 *g*, 10 min, 4°C), washed twice with 150 mM NaCl and resuspended in the same solution to an optical density of 0.8 at 400 nm, Then 2.4 ml of this bacterial suspension was mixed with 0.4 ml xylene and vibrated on a vortex-type agitator for 60 s. After standing for 15 min, the under layer sample (1 ml) was removed from the aqueous phase and the OD_400_ was measured. The formula H % = [(A_0_ – A) /A_0_] × 100 was used to calculate the bacterial surface hydrophobicity (H %), where A_0_ and A represent the absorbance of the aqueous phase before and after xylene extraction with xylene respectively. All results were obtained from at least three independent experiments. Unpaired *t*-tests were used to evaluate the data.

### Zeta Potential

The procedure for preparation of samples was described previously (Giaouris et al., 2009). Cells from *L. paracasei* L9 and L9mleS^-^ in CDMM were harvested at 12 h by centrifugation (7000 *g*, 10 min, 4°C), washed twice with 1.5 mM NaCl and resuspended in the same solution at an OD_400_ of 0.6. The electrophoretic mobility was measured with a Nano Particle Analyzer (Nano Particle Analyzer SZ-100, HORIBA Scientific, Japan). All results were obtained from at least three independent experiments. One-way analysis of variance (ANOVA) tests were used to evaluate the data.

## Results

### Global Transcriptomic Analysis of the Bile Stress Response in *L. paracasei* L9

**FIGURE 1:**
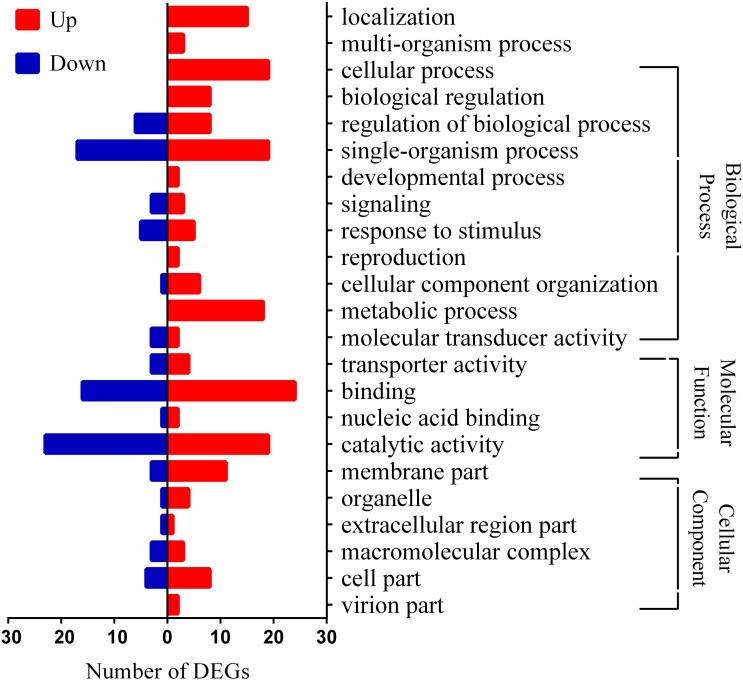
Distribution of differentially expressed genes (DEGs) in *Lactobacillus paracasei* L9 under bile stress. Bars indicate up (red) and down (blue) regulated genes (log_2_FoldChange > 1.5) in the presence of 0.13% Ox-bile.

*Lactobacillus paracasei* L9 was initially cultivated with different Ox-bile concentrations (0.1, 0.13, 0.15 and 0.2%, w/v). The growth rate was reduced by approximately 50% in the presence of 0.13% (w/v) Ox-bile (**Supplementary Figure S1**). Therefore, 0.13% bile salts was used for further studies of bile stress response in *L. paracasei* L9. The Illumina Hiseq platform was used to investigate transcriptome level changes in *L. paracasei* L9 under bile salts stress. All the raw data could be downloaded from GEO with the Accession No. GSE108713. Correlation of gene expression level between three independent biological replicates was shown by the Pearson correlation coefficient (**Supplementary Figure S2**). The number of raw and clean reads used in this study is listed in the **Supplementary Table S3**, and the information of reads mapping to the reference genome is shown in the **Supplementary Table S4**. Data analysis showed a total of 1184 differential expressed genes comprising 587 up-regulated and 597 down-regulated genes (**Supplementary Table S5**). The GOseq R package (Release2.12) was used to assign the gene ontology (GO) terms to the DEGs. Genes involved in the response to bile stress were selected for further analysis according to following standards: (1) log_2_FoldChange > 1.5; (2) statistically significant level *P* < 0.05. Finally, the transcription of 104 genes was detected to be related to bile stress, which comprised 58 up-regulated genes and 46 down-regulated genes (**Supplementary Table S6**). The putative functions of these genes were classified in different categories grouped by GO (**Figure 1**).

#### Bile Stress Expands Carbon Source Utilization Profile and Changes Carbohydrate Metabolism Pattern in *L. paracasei* L9

Genes LPL9_0432 and LPL9_0433 involved in a mannose/fructose-inducible phosphotransferase system (PTS) were 2.98 and 7.29-fold up-regulated, respectively, after exposure to bile. Meanwhile, the downstream gene (LPL9_0434) encoding the beta-fructosidase was up-regulated 2.18-fold. This beta-fructosidase can degrade the fructose polymer levan to fructose which can be transported through the mannose/fructose PTS system (Mazé et al., 2004; Monedero et al., 2007). In addition, the gene (LPL9_2760) encoding alpha-glucosidase was 3.21-fold up-regulated. Alpha-glucosidase usually extracellularly breaks down starch and disaccharides to glucose. The gene (LPL9_1931) encoding PTS system cellobiose-specific IIB component was 3.28-fold increased after exposure to bile salts. Up-regulation of cellobiose-specific EIIA transporter was also detected in *L. johnsonii* under bile stress (Lee et al., 2012). Some oligosaccharides such as fructo-oligosaccharides are considered as prebiotics because they are non-digestible food ingredients, but they may be selective substrates for potentially probiotics in the colon. The presence of bile could function as a signal for gut entry and stimulate *L. paracasei* L9 to get better adapt to the available carbon sources in the intestinal environment.

As for glucose degradation, only the gene (LPL9_1849) encoding phosphoglycerate mutase was 3.83-fold up-regulated. However, D-xylulose-5P phosphoketolase (LPL9_0177) in the hexose monophophate pathway (HMP) was 3.12-fold down-regulated at the transcriptome level. This enzyme catalyzes the conversion of xylulose-5P to acetyl-P and glyceraldehyde-3-phosphate (GAP) (Mozzi and Vignolo, 2010). Another critical enzyme in HMP, glucose-6-P dehydrogenase (LPL9_0242) was also decreased 1.52-fold under bile stress. HMP, which was repressed in response to bile in *L. paracasei* L9, mainly generates reducing power and metabolic intermediates for biosynthetic processes. These results suggest that the demand of reducing power and metabolic intermediates might be reduced as the growth of bacteria was slowed down under bile salt stress. In addition, gene (LPL9_1666) encoding a zinc-containing alcohol dehydrogenase was 2.93-fold up-regulated. In lactic acid bacteria, alcohol dehydrogenase is involved in the mixed-acid fermentation which facilitates the conversion from acetaldehyde to ethanol with the reduction of NAD^+^ to NADH (Mozzi and Vignolo, 2010). It has been reported that ethanol was produced at a higher level by the bile-resistant derivative of *B. animalis* IPLA 4549 (Ruas-Madiedo et al., 2005). In the mixed-acid fermentation, the formation of formic acid from pyruvate instead of lactic acid could yield an extra ATP, and subsequently enable the cell to recycle NAD^+^ through the generate of ethanol (Ballongue, 1998). Taken together, L9 may shift its carbohydrate metabolism to mixed-acid fermentation and then generate extra energy to enhance bile salt tolerance under bile stress.

#### Effect of Bile Stress on Amino Acids and Peptide Metabolism Processes

The *opp* operon encoding an ABC oligopeptide transport system (*opp*A, *opp*B, *opp*C, *opp*D, *opp*F) was approximately threefold up-regulated. Opp transporters are membrane-associated five-protein complexes (oppABCDF) of the ATP-binding cassette involved in uptake of di- and tripeptides (Higgins, 1992). Homologous overexpression of *opp*A has been shown to increase the tolerance to Ox-bile in *L. salivarius* Ren and confer specific resistance to taurine-conjugated bile salts, suggesting the envelope-located protein OppA may be capable of binding taurine-conjugated bile salts and then reducing the entry of these toxic compounds (Wang et al., 2015). Gene encoding a putative peptide ABC transporter was also strongly up-regulated under salt stress in *L. paracasei* ATCC 334 (Palud et al., 2018). In addition, the branched-chain amino acid (BCAA) aminotransferase (LPL9_2126), which participated in the last step of the synthesis of L-Leucine, L-Valine and L-Isoleucine, was increased 5.56-fold at transcriptional level. BCAA could form high hydrophobic structure in proteins which may resist bile salt, and similar results have also been reported in *Bifidobacterium longum* (Sánchez et al., 2005).

Several genes involved in the biosynthesis of L-lysine were up-regulated (LPL9_0091, LPL9_0086, LPL9_1309), among which the gene encoding succinyl-diaminopimelate desuccinylase (LPL9_1309, 4.69-fold) was the most significant. Meanwhile, *Lys*X (LPL9_2970) and *Lys*Y (LPL9_2971) belonging to the putative lysine ABC transport system were up-regulated 2.06 and 2.97-fold, respectively. The lysX-lysY system is involved in transporting lysine and is necessary for phosphatidylglycerol (PG) lysinylation (Rodionov et al., 2003; Maloney et al., 2011), which has been found to play a role in the resistance to cationic antimicrobial peptides (CAMPs) (Peschel et al., 2001). Meanwhile, genes encoding the biosynthesis of lysine and proteins related to lysinylation were up-regulated under bile stress in *Lactobacillus rhamnosus* GG. This modulation of bacterial surface may improve the resistance to bile by altering the surface charge to repulse cationic bile compounds (Koskenniemi et al., 2011).

#### Effect of Bile Stress on Transmembrane Transport in *L. paracasei* L9

Genes (LPL9_1281, LPL9_1282) encoding a multidrug ABC transport system were all up-regulated approximately 3.5-fold. Genes LPL9_1667 and LPL9_1668 encoding an uncharacterized ABC transport system were up-regulated about 15-fold in the bile stress response. It has been reported that multidrug transporters could export a wide range of diverse cytotoxic drugs including bile salts through cell membranes (Piddock, 2006; Whitehead et al., 2008; Pfeiler and Klaenhammer, 2009). In addition, gene (LPL9_1477) encoding a FtsE family cell division transporter was decreased 3.46-fold. FtsE is a cytoplasmic ATPase locating to the septal ring, and plays a role in cell division together with the integral membrane protein FtsX (Schmidt et al., 2004). The upstream gene (LPL9_1476) encoding a periplasmic-binding component of an ABC transporter was also 3.28-fold repressed, which probably functions related to FtsX. Recent research showed that FtsEX may be an important regulator of peptidoglycan hydrolases at the division site (Yang et al., 2011) and cells would divide poorly in the absence of FtsEX, resulting in increased cell length (Arends et al., 2009).

#### Analysis of Transcription Factors Involved in Bile Stress

A TetR family transcription factor (LPL9_0057) was 8.02-fold up-regulated, and the gene (LPL9_0056) in the same operon encoding a mycobacterial membrane protein large (MMPL) family transporter was 5.05-fold up-regulated in response to bile salt. In another operon, a TetR regulator (LPL9_1280) was 4.39-fold up-regulated and the downstream genes (LPL9_1281, LPL9_1282) encoding a multidrug ABC transporter system were also 3.95 and 3.69-fold up-regulated, respectively. The MMPL family transporters and multidrug transporters of the resistance-nodulation-cell division (RND) transporters superfamily, are usually regulated by TetR family members (Goldberg et al., 1999) and play a role in antibiotic resistance (Cuthbertson and Nodwell, 2013). The genes *bre*A and *bre*B encoding efflux pumps of the RND superfamily, were tightly controlled by the TetR family regulator BreR, and were also induced in the presence of cholate, deoxycholate, or chenodeoxycholate in *Vibrio cholerae* (Cerda-Maira et al., 2008). Another regulator (LPL9_0083) belonging to the Xre family was 4.39-fold up-regulated in detected transcripts. In *Lactobacillus acidophilus*, inactivation of the gene encoding a XRE family-like protein resulted in a more sensitive phenotype under low pH stress (Azcarate-Peril et al., 2004). Because the protonated form of bile salts could lead to intracellular acidification in a manner similar to organic acids (Smet et al., 1995). It is speculated that this Xre family regulator may be involved in bile resistance by overcoming intracellular acidification caused by bile salt in *L. paracasei* L9.

#### Response of Membrane Proteins in Bile Stress

Three genes encoding membrane proteins were up-regulated in response to bile stress, which are located in the same operon. Gene LPL9_0968 encoding a PspC-domain containing protein was 9.68-fold up-regulated, gene LPL9_0969 encoding a hypothetical protein was 10.34-fold up-regulated and gene LPL9_0970 encoding a phage holin family protein was 23.76-fold up-regulated. The PspC (phage shock protein C) is involved in extra cytoplasmic stress such as filamentous phage infection, mislocalization of some envelope proteins, extremes of temperature, osmolarity or ethanol concentration (Model et al., 1997). In *Lactobacillus reuteri*, a homolog of the phage shock transcriptional regulator PspC was reported to be related to bile stress (Whitehead et al., 2008). These membrane proteins may protect cells from environmental stress by maintaining the integrity of the cytoplasmic membrane (Darwin, 2005).

In addition, the gene (LPL9_1521) encoding a hemolysin III protein was 3.80-fold up-regulated in response to bile stress in L9. A hemolysin-like protein TlyC1 was 10-fold up-regulated upon Ox-bile treatment in *B. longum* BBMN68. Heterologous expression of *tly*C1 in *L. lactis* conferred 45-fold higher resistance to sodium taurocholate and sodium taurodeoxycholate (Liu et al., 2014). Therefore, the hemolysin protein may function as a barrier to protect *L. paracasei* L9 from bile toxicity. A gene encoding the L-Ala-D/L-Glu epimerase was increased 4.04-fold in detected transcripts, which was involved in peptidoglycan turnover and recycling (Reith and Mayer, 2011). Peptidoglycan can be degraded to the dipeptide L-Ala-D-Glu and then L-Ala-D/L-Glu epimerase could convert L-Ala-D-Glu to L-Ala-L-Glu. Only the latter can be further hydrolyzed with dipeptidases (Schmidt et al., 2001). As bile salt could cause cell wall damage (Begley et al., 2005), this epimerase may increase survival under bile stress conditions through recycling the dipeptide or amino acid from peptidoglycan degradation in *L. paracasei* L9.

### L-Malic Acid Increased the Bile Tolerance of L9

**FIGURE 2:**
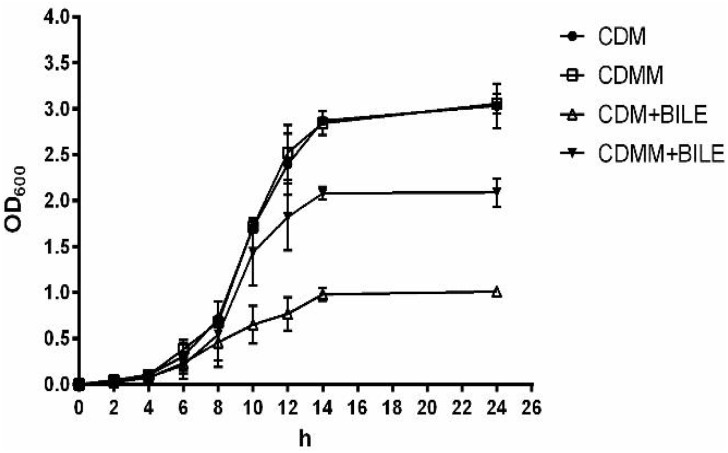
Growth of *L. paracasei* L9 in CDM (chemically defined medium) and CDMM (CDM with 2.5 mg/mL L-malic acid). Ox-bile was added at 0.2% final concentration. All results were obtained from at least three independent experiments. Error bars correspond to the standard error (SD).

Genes encoding MLE (*mle*S, LPL9_0797) and putative L-malate transporter MleT (*mle*T, LPL9_0798) were 2.60 and 3.26-fold up-regulated, respectively, in response to bile in *L. paracasei* L9. To investigate whether L-malic acid could enhance the bile resistance, *L. paracasei* was cultured in CDM with or without L-malic acid supplement. Growth of *L. paracasei* L9 was not affected by the addition of L-malic acid when cultured in the absence of Ox-bile. However, under 0.2% Ox-bile, the OD_600_ of *L. paracasei* L9 reached the maximum about 1.0 at 14 h in CDM, while the OD_600_ was 2.0 at the corresponding time in CDMM (**Figure 2**). These results demonstrated that L-malic acid in CDMM participated in the resistance to bile stress in *L. paracasei* L9.

### The Morphology of *L. paracasei* L9 in the Presence of Bile Salts

**FIGURE 3:**
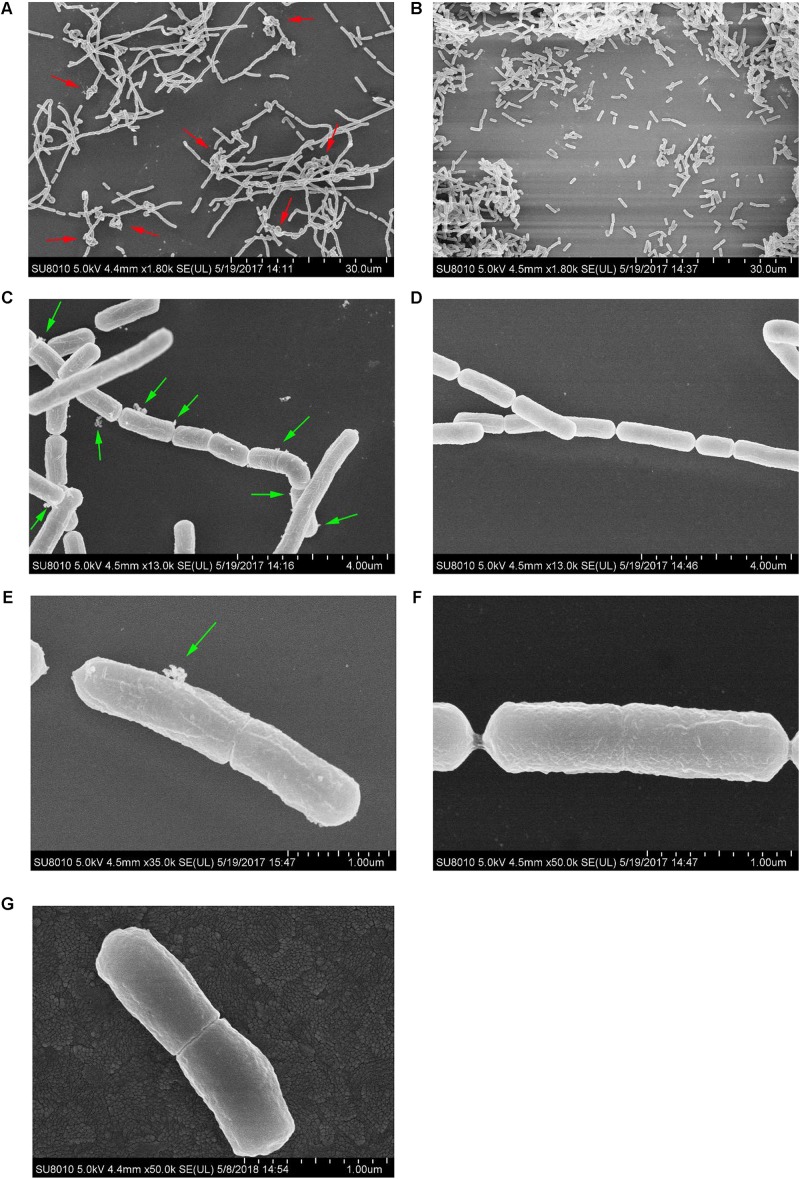
Field-emission scanning electron microscopy examination of cells of *L. paracasei* L9 treated with 0.2% Ox-bile. For three groups, the left row **(A,C,E)** was bacteria from CDM+Bile and the right **(B,D,F)** was from CDMM+Bile. **(G)** Shows bacteria from CDM without bile salt. The twist structure (red arrows) and MVs (green arrows) are indicated.

Under 0.2% bile stress, the morphology of *L. paracasei* L9 was observed using FESEM. Compared with *L. paracasei* L9 in CDMM+Bile, bacteria grown in CDM+Bile displayed a more rough and shrunken appearance (**Figures 3E,F**). Without L-malic acid, the length of a proportion of cells were increased to various degrees (**Figure 3A**), suggesting disruption in the cell division. In addition, most of the cells were not completely separated, and had a tendency to twist and clump together (**Figure 3A**), which may attribute to a change in cell surface properties. Membrane vesicles (MVs) were observed on the surface of cells under bile stress (**Figures 3C,E**). For *L. paracasei* L9 grown with L-malic acid, cells were dispersive and rod-shaped with little variation in the length. No twist or MV structures were observed in cells cultured in the medium supplemented with L-malic acid (**Figures 3B,D,F**). The morphology of bacteria grown in CDM without bile (**Figure 3G**) was more similar to bacteria grown in CDMM+Bile (**Figure 3F**). These experiments showed that L-malic acid could maintain the integrity of the cell membrane and enhance survival of *L. paracasei* L9 under bile stress.

### Malolactic Enzyme (MLE) Pathway of *L. paracasei* L9 in Bile Stress Response

**FIGURE 4:**
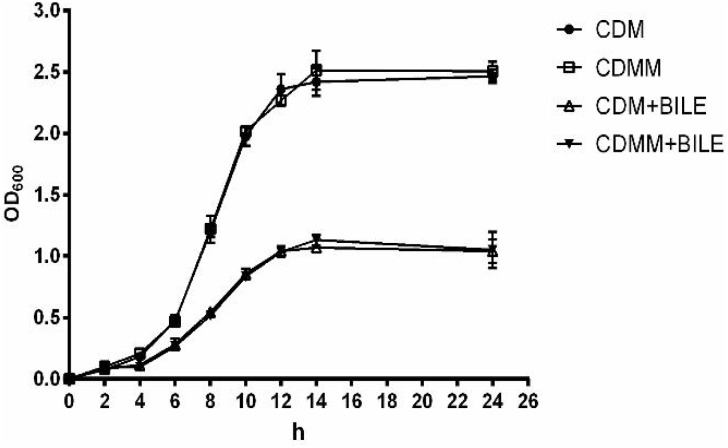
Growth of L9mleS^-^ in CDM and CDMM. Ox-bile was added at 0.2%. All results were obtained from at least three independent experiments. Error bars correspond to the standard error (SD).

To investigate if the MLE pathway is involved in the increase of bile resistance in *L. paracasei* L9, the L9mleS^-^ mutant was constructed using the suicide plasmid pUC19EM. When chromosomal DNA from the L9mleS^-^ mutant was used as template for PCR, the expected 1.5-kb product was obtained, and sequencing revealed amplification of the expected fragments of the genes *Em*^r^ and *mle*S, confirming the correct integration of pUCmleS into the chromosome of *L. paracasei* L9 by a single crossover homologous recombination event. The growth assay of L9mleS^-^ showed that growth of L9mleS^-^ was not affected by addition of L-malic acid when cultured without Ox-bile (**Figure 4**). However, the enhancement of bile tolerance in CDMM was abolished in L9mleS^-^, and both groups finally reached a maximal OD_600_ of 1.0 in CDM or CDMM under bile stress. These results demonstrated that L-malic acid is metabolized through the MLE pathway to participate in bile resistance in *L. paracasei* L9.

### Alteration of Cell Surface Properties in Response to Bile Stress

**FIGURE 5:**
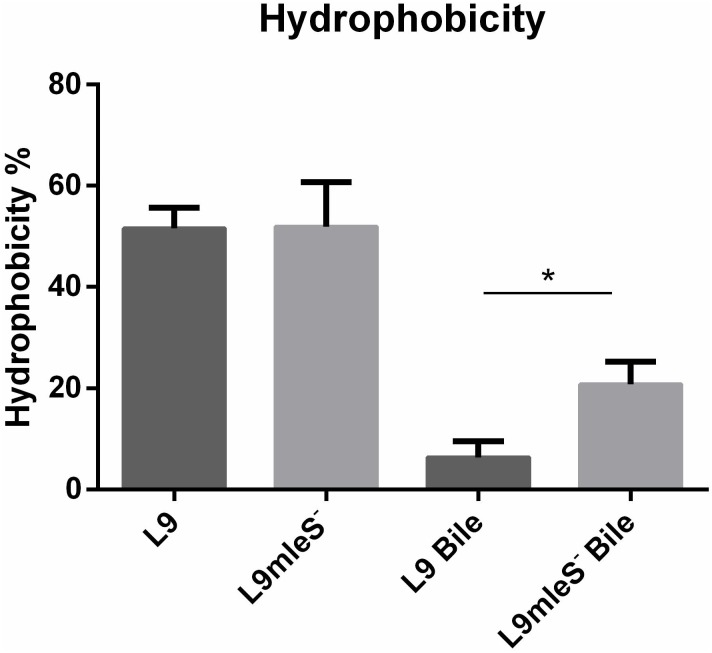
Hydrophobicity of *L. paracasei* L9 and L9mleS^-^ cultured in CDMM with or without 0.2% Ox-bile. All results were obtained from at least three independent experiments. An unpaired *t*-test was used to evaluate the data, ^∗^*P* < 0.05.

**FIGURE 6:**
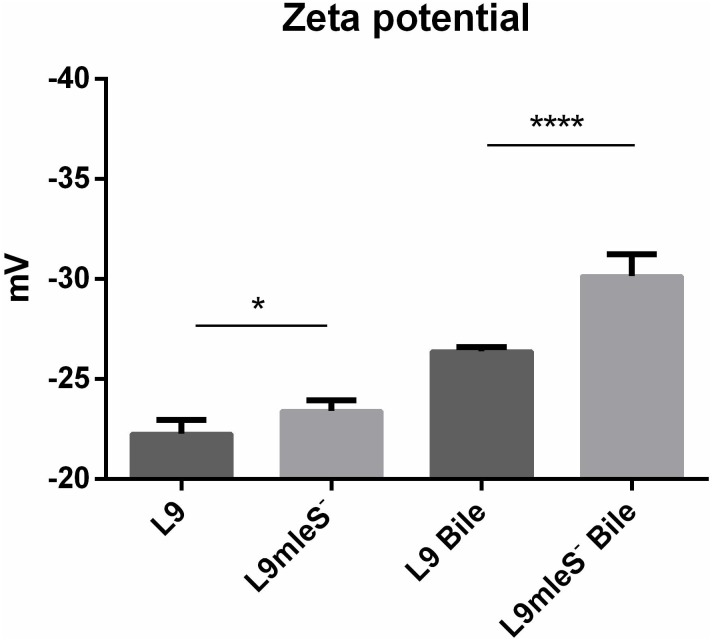
Zeta potential of *L. paracasei* L9 and L9mleS^-^ cultured in CDMM with or without 0.2% Ox-bile. All results were obtained from at least three independent experiments. One-way analysis of variance (ANOVA) test was used to evaluate the data, ^∗^*P* < 0.05, ^∗∗∗∗^*P* < 0.0001.

In the growth assays experiment, autoaggregation of L9mleS^-^ was observed at the bottom of the tube in the CDMM under 0.2% Ox-bile (**Supplementary Figure S4**). Therefore the hydrophobicity and zeta potential of *L. paracasei* strains were tested to investigate the surface properties. As shown in **Figure 5**, there is no significant difference in hydrophobicity between L9 and L9mleS^-^ cultured in CDMM without Ox-bile. However, the hydrophobicity of L9mleS^-^ was about threefold higher than that of L9 when cultured in CDMM under bile stress, which may lead to the enhancement of the autoaggregation of L9mleS^-^. In addition, there was a slight difference in the zeta potential between L9 and L9mleS^-^ cultured in CDMM (**Figure 6**), but the L9mleS^-^ strain had a significantly more negative zeta potential under bile stress. These results suggest inactivation of the MLE pathway could lead to changes in cell surface properties, which may be responsible for the decreased bile resistance in L9mleS^-^.

## Discussion

Under bile salt stress, metabolism changes at the glycolytic level which lead to enhanced energy production could be crucial for bacteria to resist bile salt. In *Lactobacillus delbrueckii* subsp. *lactis*, key enzymes in central glycolysis were over-expressed under bile stress, resulting in increased glucose utilization and lactic acid generation (Burns et al., 2010). While the bifid shunt was enhanced in *B. animalis* subsp. *lactis* and *B. longum* BBMN68 in response to bile (Sánchez et al., 2007; An et al., 2014). In this study, the carbohydrate metabolism may be shifted into the mixed-acid fermentation in *L. paracasei* L9. So, different mechanism about metabolic shifts in carbohydrate was employed to enhance energy production to resist bile stress in the different strains. As the bacterial cell membrane is the first physical target of bile salt (Begley et al., 2005), membrane proteins were often induced to resist bile stress in a variety of mechanisms, such as excreting bile salts (Ruiz et al., 2012) and acting as a barrier to protect the bacteria from bile toxicity (Liu et al., 2014). The operon (LPL9_0968-LPL9_0970) encoding membrane proteins showed the highest increase at the transcriptome level in *L. paracasei* L9, which has not been reported to be involved in the bile stress in other bacteria. Therefore, further research on these membrane proteins are needed to explain their roles in bile salt resistance.

A previous report indicated that the protonated form of bile salts could result in intracellular acidification in a way similar to organic acids (Smet et al., 1995). Generally, the genes encoding F_1_F_0_-ATPase would be up-regulated, which could maintain the internal pH under bile stress (Senior, 1990). In addition, the bile salt hydrolases (BSHs) can hydrolyze the protonated conjugated bile salts into unconjugated counterparts, and these unconjugated bile salts would prevent the pH-dropping by recapturing and exporting the co-transported proton (Smet et al., 1995). However, genes encoding BSHs do not exist in the genome of *L. paracasei* L9 and the transcription level of genes encoding F_1_F_0_-ATPase did not change under bile stress. It is noteworthy that the transcriptomic data showed that the MLE pathway was involved in bile tolerance, and this was further confirmed by genetic and physical evidence. In this pathway, L-malate acid is converted to L-lactate and carbon dioxide (CO_2_) by the MLE. As L-lactate (pKa = 3.85) has lower acidity than L-malate (pKa = 3.4), this decarboxylation contributes to alkalinization of the cytoplasm (Lemme et al., 2010). In addition, CO_2_ produced by MLE diffuses out of the cell or partially be used by carbonic anhydrase to form bicarbonate, which could further increase the intracellular pH (Papadimitriou et al., 2016). It has been reported that L-malate acid increased the survival of *L. casei* ATCC 334 under acidic conditions (Broadbent et al., 2010).

There are two gene clusters related to the metabolism of L-malate in *L. paracasei* L9. The MLE pathway consists of the gene *mle*S (LPL9_0797) encoding the MLE and gene *mle*T (LPL9_0798) encoding the putative L-malate transporter. The malic enzyme (ME) pathway in *L. paracasei* L9 consists of two diverging operons, *mae*PE (LPL9_2996, LPL9_2997) and *mae*KR (LPL9_2998, LPL9_2999). In *L. casei* BL23, growth could not be sustained by utilization of L-malate through MLE as most of lactic acid bacteria cannot metabolize lactate into the gluconeogenic pathway. In contrast, the gene *mae*E encodes a ME converting L-malate into pyruvate, which could sustain the growth of strains using L-malate as a sole carbon source. However, the ME pathway undergoes carbon catabolite repression in *L. casei* BL23 (Landete et al., 2013). In this study, it is speculated that the ME pathway is repressed by glucose in the presence of culture medium. Therefore, L-malate enhances bile tolerance mainly through the MLE pathway rather than the ME pathway in *L. paracasei* L9.

**FIGURE 7:**
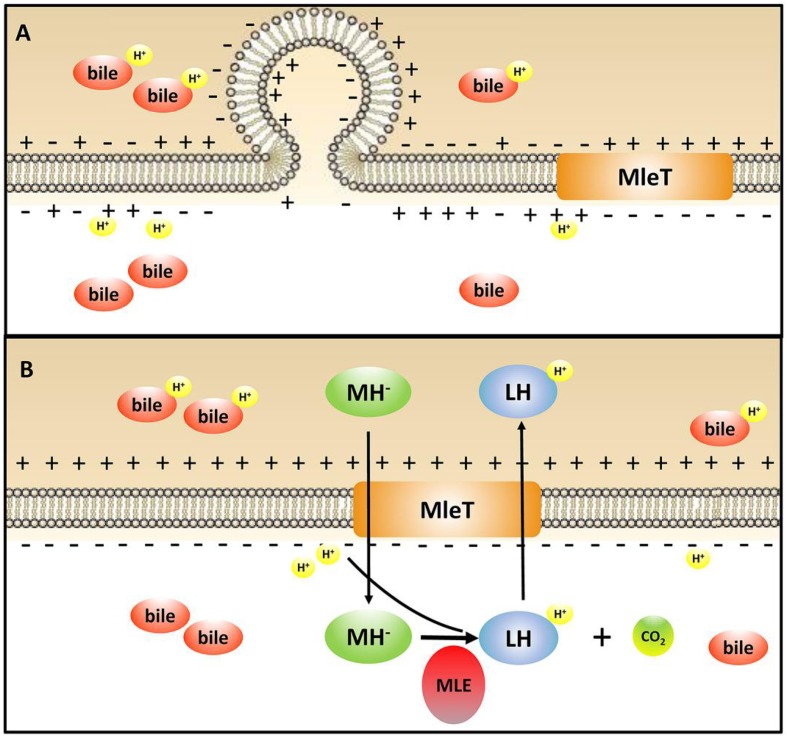
Illustration of the mechanism of MLE pathway involved in the bile stress response in *L. paracasei* L9. MH^-^, monoanionic L-malate; LH, L-lactic acid; MLE, malolactic enzyme. **(A)** *L. paracasei* L9 under bile stress without L-malic acid; **(B)** *L. paracasei* L9 under bile stress with L-malic acid.

Membrane vesicles are lumen-containing spheres formed from bulging or blebbing of the lipid-bilayer, which range in size from 20 to 500 nm in diameter, and can be found in eukaryotes, archaea, and bacteria (Deatherage and Cookson, 2012). Under different environmental stresses, formation of MVs is a common phenomenon in Gram-negative bacteria (Baumgarten et al., 2012). A molecular model of MVs formation was proposed whereby changes of electric charge on the lipopolysaccharide (LPS) caused charge-to-charge repulsion and membrane instability, and subsequently resulted in outward membrane blebbing and trapping of periplasmic components within MVs (Kadurugamuwa and Beveridge, 1995). In particular, MVs have also been observed in Gram-positive bacteria including lactic acid bacteria. MVs were observed in *L. plantarum* (Bron et al., 2004) and *B. animalis* ssp. *lactis* (Ruiz et al., 2007) under bile stress. Deformation or disruption of the cell wall was crucial for MVs to release (Brown et al., 2015), and MVs could occur when the cell wall undergoes thinning during daughter cell budding (Kopecka et al., 2000). In addition, cells with MVs were found to have a more hydrophobic surface in *Pseudomonas putida* (Baumgarten et al., 2012). Bile salts could disturb membrane integrity, which causes the leakage of protons and further dissipates the transmembrane pH and electrical potential (Kurdi et al., 2006). Based on these studies, we speculate bile salts stress could result in charge repulsions between phospholipid molecules and the bulge of membrane to generate MVs. Then the damaged cell wall may facilitate the release of MVs, which was verified by the higher hydrophobicity in L9mleS^-^ grown in CDMM under bile stress (**Figure 6**). Uptake of L- malic acid needs to be driven by a proton gradient (ΔpH) or an electrical potential (ΔΨ) (Salema et al., 1994). When bile stress disrupts the ΔΨ, internalization of L-malic acid results in the translocation of net negative charges across the membrane, which in turn creates a ΔΨ to recover the disturbance in the membrane and the zeta potential of cells could then return to a less negative level (**Figure 7**). This speculation could be verified by finding that the zeta potential of L9mleS^-^ grown in CDMM under bile stress was more negative than that of *L. paracasei* L9 (**Figure 5**). Taking all the evidence together, it is coucluded that MLE enhanced the bile tolerance not only through increasing the intracellular pH, but also by maintaining a membrane balance in *L. paracasei* L9.

## Author Contributions

YH and XM designed the study. XM and PZ performed the experiments. XM analyzed and evaluated the data. XM and YH wrote the manuscript. YH, GW, and ZZ revised the manuscript. All authors read and approved the final manuscript.

## Conflict of Interest Statement

The authors declare that the research was conducted in the absence of any commercial or financial relationships that could be construed as a potential conflict of interest.
