# Corrigendum: Global Transcriptomic Analysis and Function Identification of Malolactic Enzyme Pathway of *Lactobacillus paracasei* L9 in Response to Bile Stress

**DOI:** 10.3389/fmicb.2019.03029

**Published:** 2020-01-23

**Authors:** Xiayin Ma, Guohong Wang, Zhengyuan Zhai, Pengyu Zhou, Yanling Hao

**Affiliations:** ^1^Beijing Advanced Innovation Center for Food Nutrition and Human Health, College of Food Science and Nutritional Engineering, China Agricultural University, Beijing, China; ^2^Key Laboratory of Functional Dairy, Co-constructed by the Ministry of Education and Beijing Municipality, China Agricultural University, Beijing, China

**Keywords:** *Lactobacillus paracasei* L9, bile stress, RNA-seq, malolactic enzyme pathway, alkalinization, membrane vesicles, membrane balance

In the published article, there was an error in affiliations 1 and 2.

Instead of “Xiayin Ma^1^, Guohong Wang^1,2^, Zhengyuan Zhai^1^, Pengyu Zhou^1^ and Yanling Hao^1,2*^

1 Key Laboratory of Functional Dairy, Co-constructed by the Ministry of Education and Beijing Municipality, China Agricultural University, Beijing, China
2 The Innovation Centre of Food Nutrition and Human Health (Beijing), College of Food Science and Nutritional Engineering, China Agricultural University, Beijing, China,”

it should be “Xiayin Ma^1,2^, Guohong Wang^1,2^, Zhengyuan Zhai^1,2^, Pengyu Zhou^2^ and Yanling Hao^1,2*^

1 Beijing Advanced Innovation Center for Food Nutrition and Human Health, College of Food Science and Nutritional Engineering, China Agricultural University, Beijing, China
2 Key Laboratory of Functional Dairy, Co-constructed by the Ministry of Education and Beijing Municipality, China Agricultural University, Beijing, China.”

The authors apologize for this error and state that this does not change the scientific conclusions of the article in any way. The original article has been updated.

