# Supplementary material for: Global Transcriptomic Analysis and Function Identification of Malolactic Enzyme Pathway of Lactobacillus paracasei L9 in Response to Bile Stress
Source: Front Microbiol. 2018 Aug 23;9:1978. doi: 10.3389/fmicb.2018.01978 (PMC6119781; doi:10.3389/fmicb.2018.01978)
Supplement: Supplementary file 1 [file Table_1.docx]

**Table.S1 Bacteria strains and plasmids used in this study**

| **Strains and plasmids** | **Relevant characteristics** | **Source or reference** |
| --- | --- | --- |
| **Strains** | | |
| *L. paracasei* L9 | Host strain | Isolated from the feces of healthy  Yang *et al*., 2015 |
| *E.coli* DH5α | F^-^ϕ80d*lac*ZΔM15, Δ (*lacZYA–argF*) U169, *deoR, recA1, endA1, hsdR17* (r_K_^-^, m_K_^-^), *phoA, supE44, λ^-^ , thi-1, gyrA96, relA1.* Host strain for pUC vectors | TIANGEN |
| L9mleS^-^ | *L. paracasei* L9 with *mle*S gene interrupted | This work |
| **Plasmids** | | |
| pUC19EM | Suicide plasmid carried a Em^R^ cassette, derivative of pUC19 Amp^R^, Em^R^ | Yang *et al*., 2017 |
| pUCmleS | pUC19EM containing partial sequence of *mle*S | This work |
