# Supplementary material for: Global Transcriptomic Analysis and Function Identification of Malolactic Enzyme Pathway of Lactobacillus paracasei L9 in Response to Bile Stress
Source: Front Microbiol. 2018 Aug 23;9:1978. doi: 10.3389/fmicb.2018.01978 (PMC6119781; doi:10.3389/fmicb.2018.01978)
Supplement: Supplementary file 2 [file Table_2.docx]

| Primer | Sequence (5’-3’) | | | Restriction enzymes | Purposes | |
| --- | --- | --- | --- | --- | --- | --- |
| LPL9_M0797F | | CTAGTCTAGACGAAAATTACAGTGCGCT | *Xba*I | | | Amplify the 650bp fragment of *mle*S |
| LPL9_M0797R | | CCGGAATTCCTTCAGGACTTAAGCCT | *Eco*RI | | |  |
| 0797D-R | | GATGAAAATCGATGCTGGCAAGGCG |  | | Confirm the integration of pUCmleS | |
| EM-F | | CAAGGCAATCTGCCTCCTCATCCTC |  | |  |  |

**Table S2 Primers used in this study**
