# Supplementary material for: Global Transcriptomic Analysis and Function Identification of Malolactic Enzyme Pathway of Lactobacillus paracasei L9 in Response to Bile Stress
Source: Front Microbiol. 2018 Aug 23;9:1978. doi: 10.3389/fmicb.2018.01978 (PMC6119781; doi:10.3389/fmicb.2018.01978)
Supplement: Supplementary file 6 [file Table_6.DOCX]

**Table S6 Identification of differentially expressed genes in *L. paracasei* L9 under bile stress.**

| Category | Gene ID | Gene  Name | | Putative Function | Log_2_Fold_change | | | |
| --- | --- | --- | --- | --- | --- | --- | --- | --- |
| Carbohydrate metabolic | LPL9_1849 |  | | phosphoglycerate mutase  family 2 | | | 1.94 | |
|  | LPL9_2916 |  | | zinc-containing alcohol dehydrogenase/quinone oxidoreductase [NADPH] | | | 1.81 | |
|  | LPL9_0798 |  | | malate permease | | | 1.70 | |
|  | LPL9_2760 |  | | Alpha-glucohydrolase | | | 1.68 | |
|  | LPL9_1544 |  | | aldose 1-epimerase | | | 1.58 | |
|  | LPL9_1666 |  | | zinc-containing alcohol dehydrogenase/quinone oxidoreductase [NADPH] | | | 1.55 | |
|  | LPL9_0177 |  | | phosphoketolase | | | -1.65 | |
|  | LPL9_1176 |  | | beta-glucosidase/6-phospho-beta-glucosidase/beta-galactosidase | | | -1.66 | |
|  | LPL9_0831 |  | | Aldehyde-alcohol dehydrogenase | | | -1.70 | |
| Transporter | LPL9_1667 |  | | ABC transporter permease | | | 3.93 | |
|  | LPL9_1668 |  | | ABC transporter ATP-binding protein | | | 3.62 | |
|  | LPL9_0433 | fosX | PTS sugar transporter LevX protein | | | 2.87 | |  |
|  | LPL9_0598 |  | EpsG protein  glycosyl transferase family 2 | | | 2.78 | |  |
|  | LPL9_1419 |  | MFS transporter | | | 2.47 | |  |
|  | LPL9_1281 |  | multidrug ABC transporter ATP-binding protein | | | 1.98 | |  |
|  | LPL9_1282 |  | multidrug ABC transporter ATP-binding protein | | | 1.89 | |  |
|  | LPL9_1962 |  | ABC-type antimicrobial peptide transport system | | | 1.81 | |  |
|  | LPL9_1931 |  | PTS sugar transporter subunit IIB | | | 1.71 | |  |
|  | LPL9_0432 |  | | PTS fructose transporter subunit IID | | | 1.58 | |
|  | LPL9_2016 |  | ABC transporter ATP-binding protein | | | 1.55 | |  |
|  | LPL9_1470 |  | multidrug ABC transporter ATP-binding protein | | | -1.51 | |  |
|  | LPL9_2540 |  | phosphonate ABC transporter ATP-binding protein | | | -1.53 | |  |
|  | LPL9_2541 |  | phosphonate ABC transporter permease protein PhnE | | | -1.55 | |  |
|  | LPL9_1343 |  | ABC transporter permease | | | -2.27 | |  |
|  | LPL9_3017 |  | PTS mannitol transporter subunit IICB | | | -2.32 | |  |
|  | LPL9_2680 |  | Multidrug ABC transport ATP-binding protein | | | -2.83 | |  |
|  | LPL9_1342 |  | ABC-2 type transporter | | | -3.06 | |  |
| Amino acid transport and metabolism | LPL9_2126 |  | branched chain amino acid aminotransferase | | | 2.48 | |  |
|  | LPL9_1309 |  | acetylornithine deacetylase  Arginine biosynthesis | | | 2.23 | |  |
|  | LPL9_2937 |  | serine protease | | | 1.74 | |  |
|  | LPL9_0086 | dapB | 4-hydroxy-tetrahydrodipicolinate reductase | | | 1.73 | |  |
|  | LPL9_0091 |  | aspartokinase | | | 1.68 | |  |
|  | LPL9_2971 |  | peptide ABC transporter ATP-binding protein LysY | | | 1.57 | |  |
|  | LPL9_1911 | clpX | ATP-dependent Clp protease ATP-binding subunit ClpX | | | 1.57 | |  |
|  | LPL9_2946 | argH | argininosuccinate lyase | | | -1.51 | |  |
|  | LPL9_t028 |  | tRNA-Leu | | | -1.63 | |  |
|  | LPL9_2728 |  | aminotransferase | | | -1.66 | |  |
|  | LPL9_2945 | argG | argininosuccinate synthase | | | -1.68 | |  |
|  | LPL9_1477 | glnQ | ABC transporter ATP-binding protein | | | -1.71 | |  |
|  | LPL9_1476 |  | Glutamine ABC transporter | | | -1.79 | |  |
|  | LPL9_t044 |  | tRNA-Asn | | | -1.82 | |  |
|  | LPL9_t047 |  | tRNA-Asn | | | -2.43 | |  |
| Peptide transport and metabolism | LPL9_0488 |  | dipeptide epimerase | | | 2.01 | |  |
|  | LPL9_2155 |  | oligopeptide ABC transporter permease OppB | | | 1.92 | |  |
|  | LPL9_2154 |  | oligopeptide ABC transporter permease OppC | | | 1.81 | |  |
|  | LPL9_2156 |  | oligopeptide ABC transporter permease OppA | | | 1.78 | |  |
|  | LPL9_2153 |  | oligopeptide ABC transporter permease OppD | | | 1.65 | |  |
|  | LPL9_2200 |  | acetyl-coenzyme A carboxylase carboxyl transferase subunit beta | | | -1.71 | |  |
|  | LPL9_2207 |  | 2-nitropropane dioxygenase  FabK | | | -1.99 | |  |
|  | LPL9_2201 |  | acetyl-CoA carboxylase biotin carboxylase subunit | | | -2.17 | |  |
| Lipid transport and metabolism | LPL9_2208 |  | acyl carrier protein | | | -2.26 | |  |
|  | LPL9_2204 |  | beta-ketoacyl-[acyl-carrier-protein] synthase II  FabF | | | -2.35 | |  |
|  | LPL9_2209 |  | 3-oxoacyl-ACP synthase  FabH | | | -2.40 | |  |
|  | LPL9_2205 |  | 3-ketoacyl-ACP reductase  FabG | | | -2.49 | |  |
|  | LPL9_2202 |  | beta-hydroxyacyl-ACP dehydratase  FabZ | | | -2.54 | |  |
|  | LPL9_2211 |  | beta-hydroxyacyl-ACP dehydratase FabZ | | | -2.63 | |  |
|  | LPL9_2203 |  | acetyl-CoA carboxylase biotin carboxyl carrier protein subunit | | | -2.77 | |  |
|  | LPL9_2206 |  | Malonyl CoA-acyl carrier protein transacylase  FabD | | | -2.99 | |  |
| Transcription  regulation | LPL9_0057 |  | TetR family transcriptional regulator | | | 3.00 | |  |
|  | LPL9_1280 |  | TetR family transcriptional regulator | | | 2.13 | |  |
|  | LPL9_0083 |  | XRE family transcriptional regulator | | | 1.89 | |  |
|  | LPL9_1607 |  | transcriptional regulator | | | 1.52 | |  |
|  | LPL9_1340 |  | two-component sensor histidine kinase | | | -1.57 | |  |
|  | LPL9_1341 |  | Two-component system response regulator | | | -2.01 | |  |
|  | LPL9_2210 |  | MarR family transcriptional regulator | | | -2.27 | |  |
|  | LPL9_0316 |  | thiamin-regulated hydroxymethylpyrimidine ECF transporter | | | -2.39 | |  |
| Replication,  transcription and  translation | LPL9_2588 |  | 50S ribosomal protein L29 | | | -1.56 | |  |
|  | LPL9_1053 |  | late competence protein ComGB | | | -1.82 | |  |
|  | LPL9_2641 | cshA | DEAD-box ATP-dependent RNA helicase CshA | | | -1.83 | |  |
|  | LPL9_2579 | rpmD | 50S ribosomal protein L30 | | | -2.00 | |  |
| Membrane protein | LPL9_0970 |  | phage holin family protein | | | 4.57 | |  |
|  | LPL9_0968 |  | PspC domain-containing protein | | | 3.27 | |  |
|  | LPL9_1521 |  | hemolysin III | | | 1.92 | |  |
|  | LPL9_2598 |  | phosphate-starvation-inducible protein PsiE | | | 1.67 | |  |
|  | LPL9_0265 |  | membrane protein  LabL protein | | | -1.56 | |  |
|  | LPL9_0501 |  | pilus protein | | | -1.66 | |  |
|  | LPL9_0503 |  | class C sortase | | | -2.01 | |  |
| Function unknown | LPL9_0969 |  | hypothetical protein | | | 3.37 | |  |
|  | LPL9_2561 |  | hypothetical protein | | | 2.94 | |  |
|  | LPL9_0747 |  | hypothetical protein | | | 2.56 | |  |
|  | LPL9_0056 |  | hypothetical protein | | | 2.34 | |  |
|  | LPL9_0157 |  | hypothetical protein | | | 2.24 | |  |
|  | LPL9_0739 |  | hypothetical protein | | | 1.91 | |  |
|  | LPL9_0657 |  | hypothetical protein | | | 1.89 | |  |
|  | LPL9_2299 |  | hypothetical protein | | | 1.83 | |  |
|  | LPL9_1615 |  | hypothetical protein | | | 1.80 | |  |
|  | LPL9_0905 |  | DUF4811 domain-containing  protein | | | 1.68 | |  |
|  | LPL9_0723 |  | hypothetical protein | | | 1.62 | |  |
|  | LPL9_0585 |  | hypothetical protein | | | 1.51 | |  |
|  | LPL9_2031 |  | hypothetical protein | | | -1.51 | |  |
|  | LPL9_2642 |  | hypothetical protein | | | -1.54 | |  |
| General stress  response | LPL9_2329 | groES | co-chaperone GroES | | | 1.84 | |  |
|  | LPL9_1891 |  | Anaerobic ribonucleoside-  triphosphate reductase  activating protein | | | 1.70 | |  |
|  | LPL9_1542 | hslV | HslU--HslV peptidase  proteolytic subunit HslV | | | 1.60 | |  |
|  | LPL9_1543 |  | HslU--HslV peptidase  proteolytic subunit HslU | | | 1.53 | |  |
|  | LPL9_1783 | xseB | exodeoxyribonuclease VII  small subunit | | | -1.74 | |  |
| Others | LPL9_1418 |  | Tellurite resistance  protein TelA | | | 2.31 | |  |
|  | LPL9_0364 |  | Beta-lactamase | | | 1.80 | |  |
|  | LPL9_0487 |  | class C beta-lactamase | | | 1.75 | |  |
|  | LPL9_0319 | thiM | hydroxyethylthiazole kinase | | | -1.80 | |  |
|  | LPL9_0013 |  | cytochrome ubiquinol oxidase subunit I | | | -2.66 | |  |
