# Supplementary material for: Global Transcriptomic Analysis and Function Identification of Malolactic Enzyme Pathway of Lactobacillus paracasei L9 in Response to Bile Stress
Source: Front Microbiol. 2018 Aug 23;9:1978. doi: 10.3389/fmicb.2018.01978 (PMC6119781; doi:10.3389/fmicb.2018.01978)
Supplement: Supplementary file 7 [file Data_Sheet_1.docx]

Supplementary Material

Transcriptomic Analysis and Function Identification of Malolactic Enzyme Pathway of *Lactobacillus paracasei* L9 in Response to Bile Stress

**Xiayin Ma^2^, Guohong Wang^1, 2^, Zhengyuan Zhai^2^, Pengyu Zhou^2^, Yanling Hao^1, 2*^**

*** Correspondence:** Yanling Hao haoyl@cau.edu.cn

# Supplementary Data

## Fig. S1.tif

## Fig. S2.tif

## Fig.S3.tif

## Fig.S4.tif

## Tab S1 S2 .docx

## Tab. S3. xlsx

## Tab. S4.xlsx

## Tab S5.xlsx

## Tab S6.docx

# Supplementary Figures and Tables

**Fig. S1** The growth of *L. paracase* L9 in MRS with different Ox-bile concentrations (0.1 %, 0.13 %, 0.15 % and 0.2 %). All results were obtained by at least three independent experiments. Error bars correspond to the SD.


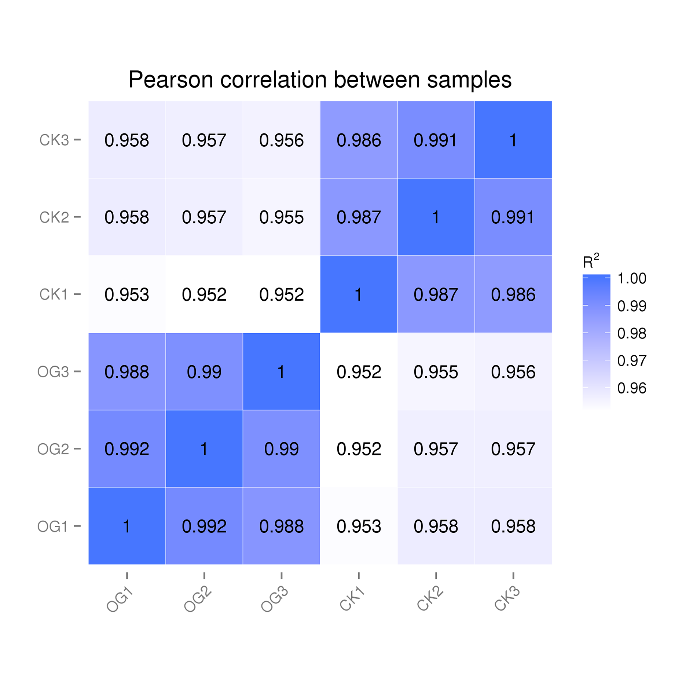


**Fig. S2** Pearson correlation between samples. CK: *L. paracasei* L9 was cultured in MRS without oxgall. OG: *L. paracasei* L9 was cultured in MRS with 0.13 % Ox-bile.


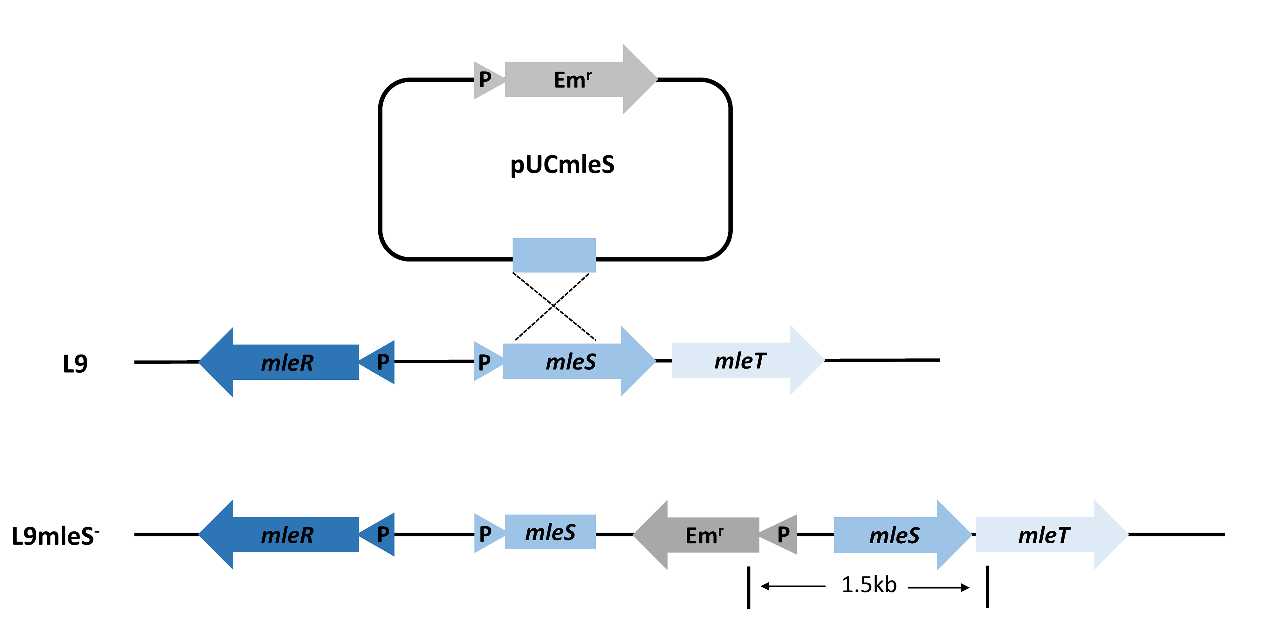


**Fig.S3** Construction of the *L. paracasei* mutant strain L9mleS^-^. Genes are represented by arrows, promoters are indicated by triangles, and the internal fragment of *mle*S is represented by a solid box. Chromosomal DNA and plasmid DNA is represented by black lines, and the 1.5Kb fragment indicates the PCR products amplified using the forward primer EM-F and the reverse primer 0797D-R.


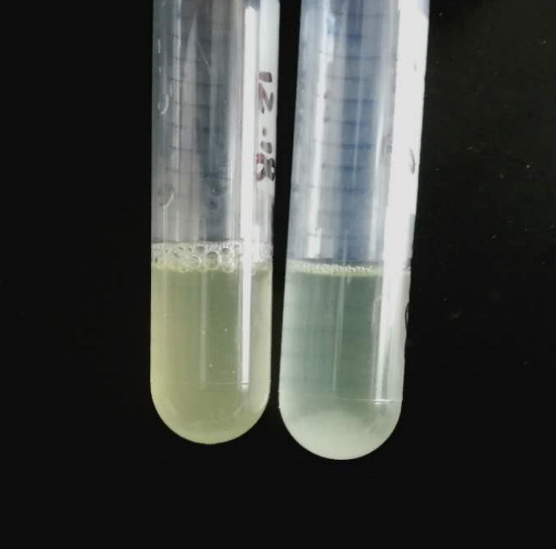


**Fig.S4** Autoaggregation of L9mleS^-^. *L. paracasei* strains were cultured in CDMM with 0.2 % Ox-bile. The left is *L. paracasei* L9 and the right is L9mleS^-^ .

**Table.S1 Bacteria strains and plasmids used in this study**

| **Strains and plasmids** | **Relevant characteristics** | **Source or reference** |
| --- | --- | --- |
| **Strains** | | |
| *L. paracasei* L9 | Host strain | Isolated from the feces of healthy  Yang *et al*., 2015 |
| *E.coli* DH5α | F^-^ϕ80d*lac*ZΔM15, Δ (*lacZYA–argF*) U169, *deoR, recA1, endA1, hsdR17* (r_K_^-^, m_K_^-^), *phoA, supE44, λ^-^ , thi-1, gyrA96, relA1.* Host strain for pUC vectors | TIANGEN |
| L9mleS^-^ | *L. paracasei* L9 with *mle*S gene interrupted | This work |
| **Plasmids** | | |
| pUC19EM | Suicide plasmid carried a Em^R^ cassette, derivative of pUC19 Amp^R^, Em^R^ | Yang *et al*., 2017 |
| pUCmleS | pUC19EM containing partial sequence of *mle*S | This work |

| Primer | Sequence (5’-3’) | | | Restriction enzymes | Purposes | |
| --- | --- | --- | --- | --- | --- | --- |
| LPL9_M0797F | | CTAGTCTAGACGAAAATTACAGTGCGCT | *Xba*I | | | Amplify the 650bp fragment of *mle*S |
| LPL9_M0797R | | CCGGAATTCCTTCAGGACTTAAGCCT | *Eco*RI | | |  |
| 0797D-R | | GATGAAAATCGATGCTGGCAAGGCG |  | | Confirm the integration of pUCmleS | |
| EM-F | | CAAGGCAATCTGCCTCCTCATCCTC |  | |  |  |

**Table S2 Primers used in this study**
